# An Assessment of the Impact of Insect Meal in Dry Food on a Dog with a Food Allergy: A Case Report

**DOI:** 10.3390/ani14192859

**Published:** 2024-10-04

**Authors:** Cinthia Gonçalves Lenz Cesar, Pedro Henrique Marchi, Andressa Rodrigues Amaral, Leonardo de Andrade Príncipe, Adrielly Aparecida do Carmo, Rafael Vessecchi Amorim Zafalon, Nelson Nobuhiro Miyamoto, Nury Aymée Collona Rodriguez Garcia, Júlio Cesar de Carvalho Balieiro, Thiago Henrique Annibale Vendramini

**Affiliations:** 1 Pet Nutrology Research Center (CEPEN Pet), Department of Animal Nutrition and Production, School of Veterinary Medicine and Animal Science, University of Sao Paulo, Pirassununga 13635-000, Brazil; cinthialenz@usp.br (C.G.L.C.); pedro.henrique.marchi@usp.br (P.H.M.); leoprincipe@usp.br (L.d.A.P.); adriellycarmo@usp.br (A.A.d.C.); rafael.zafalon@usp.br (R.V.A.Z.); balieiro@usp.br (J.C.d.C.B.); 2 Veterinary Nutrology Service, Veterinary University Hospital, School of Veterinary Medicine and Animal Science, University of Sao Paulo, Sao Paulo 05508-270, Brazil; andressa.rodrigues.amaral@usp.br; 3 Sumitomo Corporation do Brasil S/A, Sao Paulo 01311-917, Brazil; nelson.miyamoto@sumitomocorp.com (N.N.M.); nury.garcia@sumitomocorp.com (N.A.C.R.G.)

**Keywords:** canine, food, insects, protein, sustainability

## Abstract

**Simple Summary:**

Food allergy in dogs, primarily triggered by proteins, results in symptoms in the skin and gastrointestinal system. This case study focused on a 5-year-old female beagle weighing 12.4 kg, diagnosed with a food allergy with gastrointestinal manifestations, to assess the efficacy of black soldier fly larva (BSFL) meal in controlling the condition. The protocol included two nutritionally very similar diets: the control diet, with poultry by-product meal; and the BSFL diet, completely substituting the poultry by-product meal with BSFL meal. After a 12-day adaptation period to the BSFL diet, the dog maintained an adequate fecal score and showed no gastrointestinal changes. A food challenge test with the control diet induced gastrointestinal manifestations, which were reversed within two days by reintroducing the BSFL diet. The BSFL meal may be a promising option, offering improvement in gastrointestinal symptoms in dogs with food allergies. It represents a viable, cost-effective, and beneficial alternative for dogs diagnosed with food allergies.

**Abstract:**

Food allergy triggers an immune response to dietary proteins, resulting in food rejection and dermatological and gastrointestinal manifestations. The preferred therapies include diets with hydrolyzed proteins or unusual single-source proteins, with insect protein emerging as a promising option, with no reported allergic reactions in dogs with a food allergy. In this case study, the effects of including black soldier fly larva (BSFL) meal were observed in a 5-year-old spayed beagle previously diagnosed with a food allergy. The objective was to assess the potential of BSFL meal as an adjunct in treating a food allergy. As part of the protocol, two nutritionally very similar diets were used, differing only in the protein source: the control diet, with poultry by-product meal; and the BSFL diet, which completely replaced the poultry by-product meal. After a 12-day adaptation period to the BSFL diet, the dog showed no gastrointestinal changes, maintaining an adequate fecal score and no clinical signs of the disease. A challenge test with the control diet resulted in episodic gastrointestinal symptoms, which were reversed within two days by reintroducing the BSFL diet. The BSFL protein-based diet was effective in controlling the dog’s clinical signs.

## 1. Introduction

Food allergies involve various responses triggered by the immune system after ingesting foods, manifesting up to 48 hours after initial contact [1,2]. Allergic reactions to different food components can cause changes in various systems of the body, with dermatological manifestations, known as food dermatitis, being a significant concern for pet owners. Food allergy is the third most common manifestation in dogs with skin-related clinical signs, after flea bite dermatitis and atopic dermatitis [3]. According to Olivry and Mueller [4], the prevalence of dogs with food-related skin reactions is 1 to 2% among all cases, and can reach up to 24% of dermatological cases. Additionally, food allergies may manifest as gastrointestinal signs and are often characterized by vomiting, soft stools, diarrhea, flatulence, and an increased frequency of bowel movements, and are less common than dermatitis [1].

Despite the various causes, protein is one of the main allergens in dogs. According to the study by Mueller et al. [5], the protein sources most frequently associated with allergies in dogs are beef (34%), dairy products (17%), and poultry (15%). Additionally, allergies to other proteins such as lamb (5%), eggs (4%), pork (2%), and fish (2%) can also occur, although with lower incidence. Other ingredients besides proteins may affect sensitive dogs, such as wheat (13%), soy (5%), corn (4%), and rice (2%).

The preferred therapy for food allergies is utilizing diets with a single, unusual protein source or diets with hydrolyzed proteins [6]. Diagnosing food allergies involves implementing a food trial, offering foods with unusual proteins and observing symptom improvement, followed by a food challenge to observe the recurrence of symptoms and confirm the diagnosis [7].

In Europe, the term “hypoallergenic” is widely used as the main claim for pet food products containing insects [8,9]. Research on the compatibility of insect-derived proteins with allergic dogs is still a relatively unexplored area. However, there have been no documented allergic reactions in sensitive dogs and cats after consuming insect-based diets [10]. It is believed that the low allergenicity of the insect proteins is due to their novelty and the lack of familiarity of the dog’s immune system with these substances [11]. 

However, some studies have shown that humans and rats can present primary allergic reactions to arginine kinase, paramyosin, tropomyosin, and chitin after consuming mealworm beetles and crickets [12,13,14]. Additionally, a second pathway could be due to cross-reactions between different insect species and other allergenic components [15]. Thus, since the understanding of the topic is still limited, it is important to recognize that each animal organism may react differently to the novel insect proteins.

The use of black soldier fly larva (BSFL) meal can be considered a sustainable protein source, as it utilizes about 80% of its biomass, compared to traditional animal husbandry sources like beef and poultry, which utilize only about 40–60% of the animal, resulting in fewer residual by-products [16]. Additionally, it has a high feed conversion rate, ranging from 0.43 to 0.55, which surpasses the maximum ratio of 0.30 observed in poultry [17].

Beyond production and sustainability benefits, BSFL meal has a high concentration of lipids and protein, containing all the essential amino acids [18,19,20]. The fat produced from this meal is rich in fatty acids, particularly lauric acid, and has a content of omega-3 polyunsaturated fatty acids, ranging from 1% to 3.6% [21]. Regarding protein, insect meal can range between 50% and 77%, depending on the species, and contains significant amounts of minerals such as iron, magnesium, selenium, zinc, and copper [20]. Additionally, this ingredient has shown good acceptance and tolerance by dogs and other animals [16,22].

This case report aimed to observe the potential of a food containing a single protein source from BSFL meal as an adjunct treatment for a food allergy in a dog diagnosed with this condition.

## 2. Material and Methods

All experimental procedures were approved by the Ethics Research Committee for Animal Welfare of the School of Veterinary Medicine and Animal Science at the University of Sao Paulo (protocol number: 2608270723). 

## 3. Case Report

A 5-year-old spayed female dog, weighing 12.4 kg with a body condition score of 5 [23] and a muscle mass score of 3 [24], was kept up-to-date on vaccinations, deworming, and ectoparasite control. She belonged to the kennel of the Pet Nutrology Research Center (CEPEN Pet) at the Department of Animal Nutrition and Production, Veterinary Medicine and Animal Science School, University of Sao Paulo (FMVZ/USP). The dog had a history of tenesmus, diarrhea, hematochezia, and mucus in the feces, and was not on any medications or supplements.

Complementary laboratory tests were performed, including blood collection for red cell count, serum biochemistry (urea, creatinine, alanine aminotransferase, alkaline phosphatase, total proteins, albumin, globulins, cholesterol, and triglycerides), and blood glucose levels. All analyzed parameters were within the reference values for the species, which ruled out the presence of any comorbidities.

**Figure 1:**
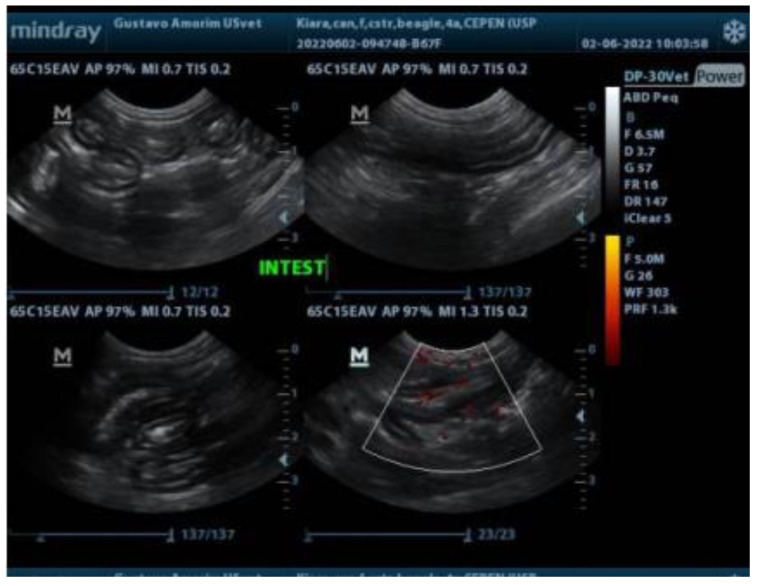
Ultrasonographic examination of a female beagle dog showing diffuse thickening of the muscular layer and generalized increased vascularization of the intestine.

An ultrasonographic examination was conducted to evaluate the gastrointestinal tract after the dog had fasted for 12 hours. In the ultrasonographic images, gas was observed in some regions along the gastrointestinal tract, and diffuse thickening of the muscular layer and generalized increased vascularization were identified (Figure 1). No abnormalities were identified in other systems.

To achieve a diagnosis, the animal was initially placed on a hypoallergenic diet containing hydrolyzed poultry, which completely resolved her symptoms and maintained stability. She was subsequently transitioned to an insect-based diet (BSFL diet), where the protein source was fully replaced with BSFL meal (Cyns, Piracicaba, São Paulo, Brazil), for a 12-day period to evaluate the diet’s effectiveness in maintaining her clinical condition. Following this, she was given a control diet, which had the same formulation as the BSFL diet but with the protein source replaced by poultry by-product meal (Table 1). This was used as a food challenge test to assess for a potential relapse of enteritis. If a relapse occurred, the BSFL diet would be reintroduced to confirm its effectiveness.

## 4. Results

**Figure 2:**
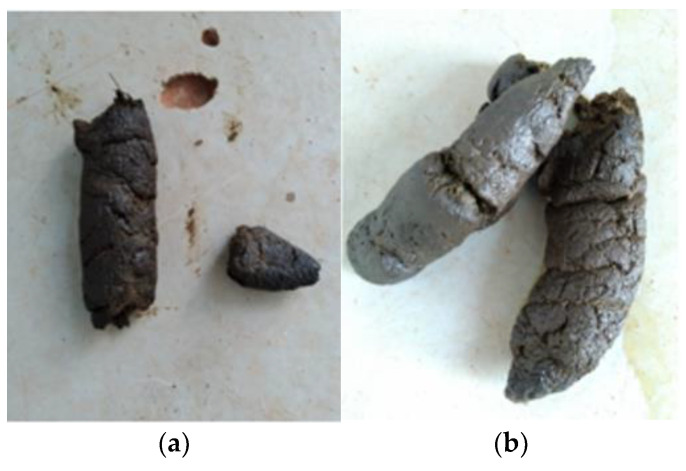
(**a**,**b**) are the patient’s feces during BSFL diet intake.

During the 12-day adaptation period to the BSFL diet, the animal’s fecal score remained adequate (Figure 2). On physical and clinical examination, no episodes of vomiting or food refusal were observed, and there were no signs of abdominal sensitivity or pain.

**Figure 3:**
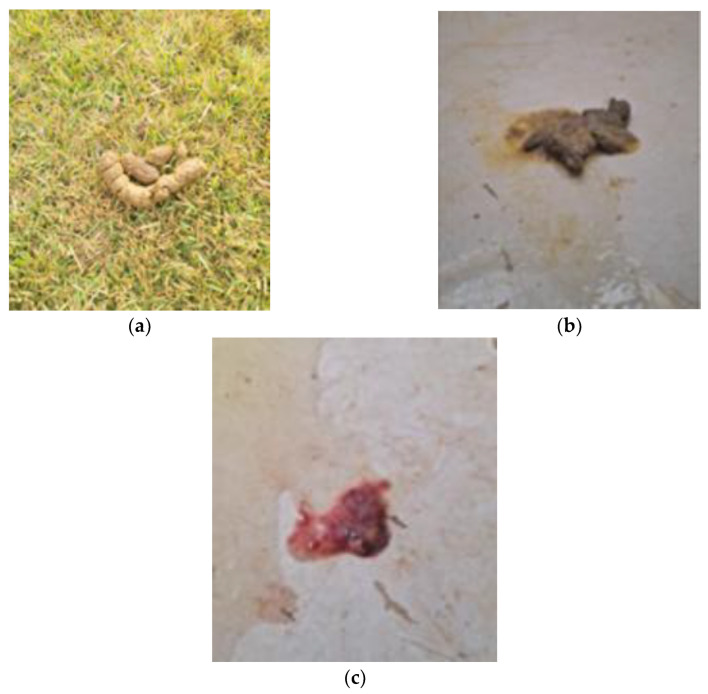
Patient’s feces during control diet intake. (**a**) Soft stool, with moist surface, but still holding form on day one; (**b**) very soft, formless and very moist feces on day three; (**c**) watery diarrhea with hematochezia on day seven.

Subsequently, a challenge test with the control diet was conducted for ten days to investigate any potential adverse effects of sensitivity in the animal. During this period, the animal exhibited episodic gastrointestinal symptoms (Figure 3), including feces with mucus and hematochezia.

**Figure 4:**
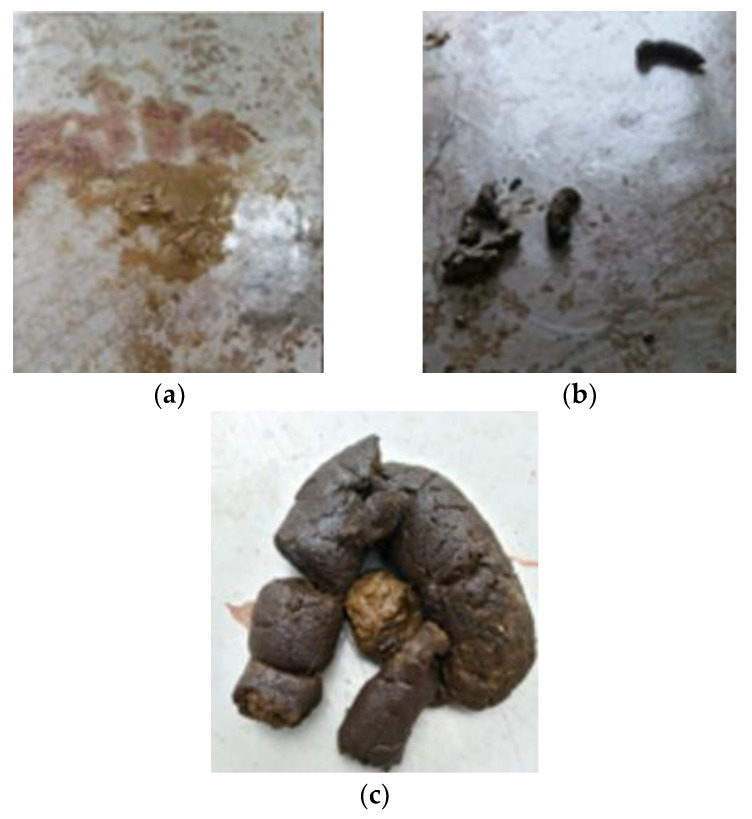
Patient’s feces after reintroduction of BSFL diet. (**a**) day zero; (**b**) day one and (**c**) day two.

As a validation of the potential of the insect meal diet, a counterproof was conducted through the reintroduction of the BSFL diet. Improvement in symptoms was observed within just two days, and after five days, there was complete remission of all clinical signs associated with food allergy (Figure 4).

## 5. Discussion

The treatment for a food allergy involves using diets composed of a single, unusual protein source for the animal or diets containing hydrolyzed proteins [6]. The diagnosis of a food allergy includes implementing a food trial, where foods with unusual proteins are offered and symptom improvement is monitored. This period is followed by a food challenge, where the reintroduction of suspected allergenic foods leads to the recurrence of symptoms, confirming the diagnosis [7].

As a novel protein source, a diet containing BSFL meal may serve as a hypoallergenic option for diagnosing and managing patients with food allergies. To confirm the effectiveness of the BSFL diet, a challenge test was conducted using a control diet with the same ingredients as the BSFL diet, but with poultry by-product meal as the protein source.

It was observed that the patient exhibited food allergy symptoms after consuming the control diet, marked by episodic gastrointestinal manifestations such as mucus in the feces and hematochezia. Since the control diet, apart from the protein source, contained the same ingredients as the BSFL diet, we can conclude that the animal was allergic to poultry protein. In contrast, the BSFL diet did not provoke any allergic reactions. It was well-tolerated, with no episodes of emesis, food rejection, sensitivity, abdominal pain, or borborygmi observed in the intestinal loops.

For insects to become a source of protein in pets’ diets, it is necessary to investigate the potential risk of adverse food reactions, including allergic reactions [13]. In Europe, since 2018, insects have been classified as food, requiring mandatory evaluation by the European Food Safety Authority (EFSA) before commercialization. The first safety assessment related to insect consumption was conducted by EFSA in 2015 [26]. 

Potential risks associated with using insects in animal or human food have been evaluated in terms of both external and internal factors. It was concluded that insect-based food ingredients could trigger an allergic response due to primary sensitization or cross-reactions [27]. Cross-reactions involve IgE cross-reactivity between insect proteins and known allergens from species within the arthropod phylum, including mites and crustaceans [28]. In a study by Premrov et al. [29], dogs allergic to mites showed cross-reactivity to proteins from yellow mealworm larva meal.

A recent study identified two variants of tropomyosin in the black soldier fly genome, with molecular structures potentially allergenic to vertebrates [30]. Tropomyosin is a major allergen associated with crustaceans and mites, which increases the likelihood of cross-reactions after the consumption of BSFL-based foods [15,31]. Additionally, another concern is the bioaccumulation of heavy metals in BSFL, mainly cadmium and mercury [32]. Bessa et al. [33] observed levels of heavy metal accumulation below the limits established for crustaceans and seafood in BSFL samples. Nevertheless, further research and the establishment of legal limits for new insect sources introduced into the human and pet markets are essential.

Therefore, this ingredient should be used cautiously in the diets of dogs with allergies. However, there are no reports of food allergies associated with the use of BSFL meal.

## 6. Conclusions

Based on this case report and the absence of allergic reactions to black soldier fly larva meal, we can conclude that foods containing this ingredient provide a promising and viable alternative for managing food allergy in dogs.

## Figures and Tables

**Table 1:** Chemical composition of experimental foods.

Item	Diets	
	Control Diet ^3^	BSFL Diet ^4^
Dry matter (%)	94.66	94.76
	Chemical composition in DM (%)	
Organic matter	93.25	92.87
Crude protein	32.26	30.79
Fat	13.91	13.24
Ash	6.65	7.13
Crude fiber	1.85	4.84
Nitrogen-free extract ^1^	45.33	44.00
Metabolizable energy ^2^ (kcal/g)^2^	4.06	4.00

BSFL = black soldier fly larva; DM = dry matter; ^1^ Nitrogen-free extract was calculated by the difference in the known macronutrient content; ^2^ Metabolizable energy was estimated according to NRC [25]; ^3^ Ingredients listed in descending order based on content: corn, poultry by-product meal, rice bran, beet pulp, fresh viscera oil, palatant 11 liters, palatant 9P, potassium chloride, seaweed meal, vitamin premix, salt, choline 60%, taurine, mineral premix, methionine 99%, antioxidant powder, L-tryptophan; ^4^ Ingredients listed in descending order based on content: corn, black soldier fly larva meal, rice bran, beet pulp, fresh viscera oil, palatant 11 liters, palatant 9P, potassium chloride, seaweed meal, vitamin premix, salt, choline 60%, taurine, mineral premix, methionine 99%, antioxidant powder, L-tryptophan.

## Data Availability

The original contributions presented in the study are included in the article, further inquiries can be directed to the corresponding author.
